# Swedish Healthcare Direct managers’ views on gender (in)equity: applying a conceptual model

**DOI:** 10.1186/s12939-019-1011-5

**Published:** 2019-07-24

**Authors:** Elenor Kaminsky, Anna T. Höglund

**Affiliations:** Department of Public Health and Caring Sciences, Box 564, SE-751 22 Uppsala, Sweden

**Keywords:** Equity in health, Conceptual model, Telephone nursing, Gender, Sweden

## Abstract

**Background:**

Although Swedish legislation prescribes equity in healthcare, inequitable healthcare is repeatedly reported in Sweden. Telephone nursing is suggested to promote equitable healthcare, making it just one call away for anyone, at any time, irrespective of distance. However, paediatric health calls reflect that male parents are referred to other health services twice as much as female parents are. Regarding equity in healthcare, telephone nurses have expressed a continuum from Denial and Defence to Openness and Awareness. To make a change, Action is also needed, within organizational frames. The aim here was thus to investigate Swedish Healthcare Direct managers’ views on gender (in)equity in healthcare through the application of a conceptual model, developed based on empirical Swedish Healthcare Direct telephone RN data, as a baseline measure at the service’s national implementation.

**Methods:**

All Swedish Healthcare Direct managers were interviewed during the period March–May 2012. They were asked how they view equitable healthcare, and how they work to achieve it. A conceptual model for attaining equity in healthcare, including Denial, Defence, Openness, Awareness and Action, was used in a deductive thematic analysis of the interview data.

**Results:**

The five model concepts – Denial; Defence; Openness; Awareness and Action – were found in a variety of combinations in the manager interviews. Denial and Defence were mentioned to a higher extent than Openness and Awareness. Several informants denied inequity, arguing that the decision support tool prevented this. However, those who primarily expressed Denial and Defence were also open to learning more on the subject. Action was only mentioned twice in the informants’ answers, and then only implicitly.

**Conclusion:**

Although a majority of the interviewed managers expressed a lack of awareness of (in)equity in healthcare, they also expressed an openness to learning more. While this may reflect a desire to show political correctness, it also points to the need for educational training in order to increase the awareness of (in)equity in healthcare among healthcare managers. Future follow up measurements will reveal if this has happened.

## Background

Swedish Healthcare Direct (SHD) is a service similar to the British NHS 111. It was launched in 2003 in four Swedish county councils, based on a belief that telephone nursing (TN) would improve the possibilities for healthcare to reach the goals of good health and care on equal terms for the whole population [1], which are stated in the Swedish Healthcare Act, 2017:30 [2]. For a decade the service gradually expanded, and was available nationwide in 2013 [3], including all counties in Sweden. Since then, among the Swedish population of 10.2 million, approximately 5.5 million calls are made annually to the approximately 33 SHD sites around the country.

Every region and county council is responsible for its geographical part of Sweden. Callers reach the service’s approximately 1500 employed SHD registered nurses (RNs) directly. This can be compared to callers to NHS 111 in the UK, who instead are first assessed by call handlers who have undergone 3 weeks of healthcare education. A decision support tool (DST) is used for the RN assessments in Sweden. In line with international literature [4–6], around 50% of the calls result in the RN giving self-care advice, while the remaining calls lead to a recommendation to visit health services, e.g., a physician or RN.

Contrary to the law, gender has been shown to play a role in the outcome of SHD calls [7, 8]. Men call health services to a lower extent than women do [7, 9–12]; and when they do call, they are advised to visit health services instead of being given self-care advice twice as often as women are [7, 8, 13, 14]. This is in line with findings regarding face-to-face encounters [15, 16], and reveals that, thus far, TN has not improved the possibility to fulfil the law’s requirements regarding healthcare on equal terms for the whole population.

Several possible reasons have been suggested for the female caller majority and the increased self-care advice outcome of their calls [7, 8]. These include: families’ own choice and responsibility [17, 18]; telephone RNs’ management of calls (based on their own and/or the organization’s criteria) and societal gender norms [11, 17]. The organization’s advertisements to certain caller groups, as well as the RNs’ call-centre-like working environment with monitored calls and time limits [19], have also been suggested as possible explanations [3].

According to gender theory, the performance of care has long been identified as a central characteristic of womanhood [20–22]; e.g., reflected in seeing nursing, childcare, and family work as female tasks. In Sweden, despite a long history of equality work and politics, women are still the main providers of care within families [23]. Hence, it could be argued that caring duties continue to be central to the social construction of women’s gendered identity. Gender norms have further distinguished between femininity, as connected to the private sphere, and masculinity, as connected to the public sphere [24–26]. SHD is somewhat of a break point on this matter, since it is a public service in which private issues are discussed. Consequently, many parameters are at play regarding gender in SHD calls. The involved SHD stakeholders are the callers, the telephone RNs, and the organization/healthcare provider, represented by the SHD managers.

## Theoretical framework

The concept of “gender” came into use in the 1970s, in order to be able to study differences between men and women beyond biological sex [27, 28]. A decade later West and Zimmerman [29] introduced the concept of “doing gender”, implying that gender is constantly being constructed in interaction between people. Initially, the focus of the theory development was the construction of femininity, addressing women’s subordination in patriarchal societies. In the 1990s, theories of masculinity were developed. Connell [30, 31] argued that femininity and masculinity are contextually and relationally constructed, implying that several forms of them can be found within the same context. Further, different forms of masculinity (and femininity) are hierarchically ordered in relation to each other in different contexts. In a Western context, “hegemonic masculinity” has been the most valued and normative form of masculinity, constructed as superior to femininity and to other forms of masculinity [30, 32]. Hegemonic masculinity might not be the most common form of masculinity – few men likely live up to its idealized images – but when constructing masculinity, men position themselves in relation to this norm. A corresponding “hegemonic femininity” has been recognized by Schippers [33]. It is seen as the most valued form of femininity and reflects men occupying dominant positions, superior to women. Hegemonic femininity has further been argued to manifest caring and acting responsibilities in families and societies [34].

Research on equity in health has been performed based on these theories [15, 16, 35]. In Sweden, the Health Care Act, 2017:30 [2], prescribes equity in health not only for the whole population, but also to everyone according to their particular needs. The World Health Organization (WHO) [36] defines equity as the “absence of avoidable, unfair, or remediable differences among groups of people, whether those groups are defined socially, economically, demographically or geographically or by other means of stratification” (http://www.who.int/topics/health_equity/en/). Further, WHO states: “‘Health equity’ or ‘equity in health’ implies that ideally everyone should have a fair opportunity to attain their full health potential and that no one should be disadvantaged from achieving this potential.”

**Fig. 1:**
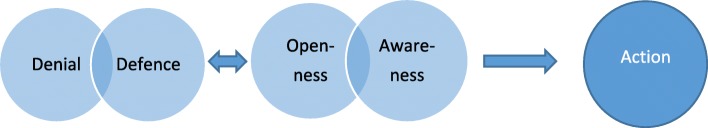
Description of the conceptual model with the four concepts as two qualitatively different blocks, but also as positions on a continuum, and with Action as a fifth concept (Höglund et al. 2018)

In a previous study, we developed a conceptual model for attaining equity in healthcare [37], since tools for assessing (in)equity in healthcare are scarce. The model emerged from empirical data on SHD telephone RNs’ views on equity in healthcare, and includes the concepts Denial, Defence, Openness, Awareness, and Action; see Fig. 1. Denial involves denying the existence of (in)equity in healthcare, while Defence involves acknowledging it but not working against it. Openness indicates a readiness to learn and develop competence regarding (in)equity in healthcare, whereas Awareness entails an expressed consciousness of the subject. Action, finally, implies the ability to recognize challenging situations and evaluate and act upon them, in order to facilitate equity in healthcare.

The model concepts are positioned on a continuum in which an individual, or a group of individuals, can move from one position to another. Individuals can thus develop and mature and, consequently, go from Denial, via Defence and Openness, to Awareness. Furthermore, to make a change and achieve equity in healthcare, Action is needed [37]. Different contexts and circumstances can possibly make an individual advance to new positions on the continuum. An alternative way of looking at the four concepts is as two qualitatively different blocks. In this case, Denial and Defence are placed on one side of the model and Openness and Awareness on the other. The model recognizes Denial and Defence as connected to a resistance to acknowledging prevailing discriminatory structures. Both positions thus describe an opposition to increased awareness of inequalities and active actions for equity. The concepts Openness and Awareness are similarly connected, as visualized in the model. They represent an awareness of today’s imperfect healthcare, in parallel with working for change regarding discriminatory structures.

A combination of the two interpretations described above is presented in our earlier study [37]. The categories Denial and Defence were argued to be qualitatively different in character from Openness and Awareness. Hence, they can be seen as two separate blocks. At the same time, the benefit of interpreting all five categories as positions on a continuum was suggested, as this allows room for development and expansion within individuals and at workplaces. Consequently, a combined model was suggested, admitting both similarity and overlap between Denial and Defence on the one hand and between Openness and Awareness on the other, but at the same time defining them as positions on a continuum. It was further argued that the model needed to include Action, in order to achieve equity in healthcare [37]. Being aware is not enough – an ability to act upon identified unequal situations is also needed. Thus, the concept of Action was added to the model, although only implicitly emerging from empirical SHD telephone RN data.

SHD telephone RNs are supervised by SHD managers, who also monitor and control their calls [38, 39]. For example, SHD managers are to ensure that the national goal of no more than a three-minute queue for callers is reached. In a previous study [18], all SHD managers stated that equitable healthcare is important. However, they indicated that a gender-equal caller population is not something SHD can do anything about. The fact that female parents make the majority of calls was regarded by most managers as a family issue. However, the managers were concerned about gender differences in outcomes of calls [7, 8].

The SHD managers’ explanations for gender inequities in Swedish TN thus varied, and seemed complex to interpret. Since the time of that study, we have developed the above-described conceptual model for attaining equity in healthcare [37]. In the present study, we apply this model to existing SHD manager interview data [18], in order to reach a deeper understanding of how SHD managers viewed the goal of equity in SHD at the time of its national implementation.

### Aim

The aim of the present study was to investigate SHD managers’ views on gender (in)equity in healthcare through the application of a conceptual model, developed based on empirical SHD telephone RN data, as a baseline measure at the service’s national implementation.

## Method

### Design

The conceptual model described in the background section was applied on manager interview data. Hence, the study used a deductive design, using qualitative interviews that were analysed in light of a conceptual model [40].

### Study participants and procedure

All SHD managers in the Swedish county councils and regions were contacted for the study (*n* = 23). They were informed about the study via e-mail. Two weeks later, all managers were contacted over the phone and all 23 agreed to participate. Individual telephone interviews were performed in Swedish during the period March–May 2012 by EK, lasting 35–70 min. The interview questions were semi-structured and concerned the managers’ views on the goals of TN work, health promotion, and gender equity in healthcare. The part on equity was introduced through a short resume of previous research results according to Kaminsky et al. [7], reporting that female parents had contacted SHD to a higher extent than male parents regarding paediatric health calls, and that the outcome of such calls differed based on parent gender. Thereafter, the following questions were asked:

- Do you recognize the figures concerning the gender (im)balance of callers to SHD?
- What are your reflections on these figures?
- Do you think they will change over the years?
- Would you like to impact these figures? If yes: How?
- What are your reflections on the results concerning gender-based differences in the outcomes of paediatric calls to SHD?

The results from the questions on gender and equity, with the conceptual model applied, are presented here. Other results are reported elsewhere [18].

Of the 23 SHD managers, 21 were women and two were men, and they were aged 40–65 years (M = 54). At the time of the study, they were responsible for all of Sweden’s SHD sites. Sixteen managers had clinical experience in TN themselves. Their experience as SHD managers varied from 0.3 to 12 years. All but two had RN degrees within a variety of specialties, and their RN experience ranged from 9 to 36 years. Among the remaining managers, one was a psychologist and one a mental health worker. Three managers were privately employed, and 20 worked within the public healthcare system. However, all SHD sites, like most Swedish healthcare, were publicly financed.

### Analysis

The interviews were recorded and transcribed verbatim. Data were analysed by both authors individually, using deductive directed content analysis as described by Hsieh and Shannon (2005). All transcripts were read and re-read, and texts answering the study aim were identified and sorted into the different concepts in the conceptual model. Hsieh and Shannon [41] highlight that “The goal of a directed approach to content analysis is to validate or extend conceptually a theoretical framework or theory” and further that “Existing theory or research can help focus the research question” [41]. This was found to be suitable to this study, with the aim of deductively studying how SHD managers view equity in healthcare in light of a conceptual model. EK performed the primary analysis. ATH read transcripts and commented on the initial coding. The final version of the analysis was discussed and consented to by both authors.

### Ethical considerations

The present study followed the ethical regulations and guidelines according to The Act concerning the Ethical Review of Research Involving Humans, SFS 2003:460 [42]. Further, it also conformed to relevant ethical principles in the World Medical Association Declaration of Helsinki [43]. All SHD managers chose to participate after being guaranteed confidentiality and informed that participation was voluntary and that they could withdraw from the study at any time, without giving a reason. The data have been handled confidentially, and the results are presented so that individuals cannot be identified.

## Results

When applying the conceptual model for attaining equity in healthcare to SHD manager interview data, all concepts of the conceptual model proved to be present in the answers. This means that the concepts Denial, Defence, Openness and Awareness, followed by Action, were all represented. In the following, each concept will be discussed and illustrated by quotes. For an overview of the frequency of expressed concepts in results, see Table 1*.*

**Table 1:**
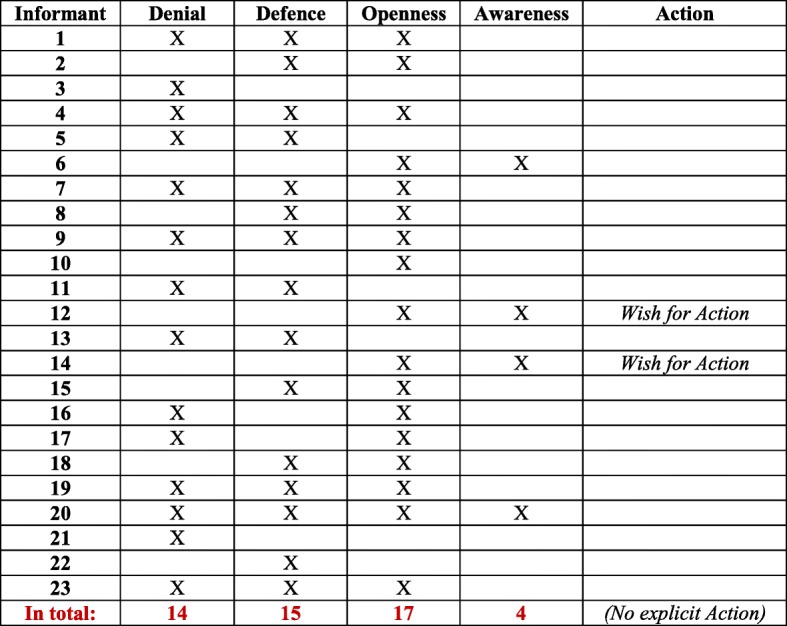
Frequency of expressed concepts (Denial/Defence/Openness/Awareness/Action) in the 23 interviews

### Denial

The first concept in the conceptual model, Denial, was present in 14 of the 23 SHD manager interviews. This concept captures a person’s denial of the existence of inequity in healthcare in general, and in telephone nursing in particular, as well as to some extent in society as a whole. The managers expressing Denial communicated that healthcare is provided equally, and that (in)equity in health is not a problem, particularly in telephone nursing. For example, two managers stated:

*No, that* [unequal outcomes of paediatric calls] *isn’t really right…we’re moving toward a more equal approach* [Informant 3]

*I don’t get the sense that there would be different handling depending on gender, or mother or father* [Informant 19]

In two interviews, Denial was the only concept represented. Further, in three interviews, Denial was expressed together with Defence. In six interviews, Denial was expressed together with Defence and Openness. Moreover, in two interviews, it was expressed together with Openness. Finally, in one interview, Denial was mentioned together with the remaining three concepts Defence, Openness, and Awareness.

### Defence

The second concept in the conceptual model, Defence, was represented in 15 of the 23 SHD manager interviews. This concept implies that a person acknowledges the existence of inequity in healthcare, but is still reluctant to work against it. Some of the managers stated that the RN assessments have to be made according to a computerized DST. They stressed that the DST assures RNs’ standardized assessments, simultaneously as they stated that exactly the same treatment for all callers is an impossible matter:

*We do after all have a decision support tool, and that’s the medical assessment we’re to make* [Informant 1]

*There’s nobody who can stand there and say we do exactly the same thing for everybody, because you know how much we’re influenced by everything under the sun* [Informant 5]

In interviews in which managers displayed Defence, it was also underlined that it must be the child’s condition that dictates the paediatric health call assessments, and not which parent is calling the SHD:

*It should naturally be the child’s condition that determines* [the assessment]*, not the person who’s calling* [Informant 4]

*Because, you know, I don’t handle a father who’s calling for his child differently from if I’ve got a mother; the assessment is made based on the advisement guidelines and what they say…Because we, you know, ask questions and we get the answers we get and, you know, we can’t question whether they’re right or true or such either…I can’t imagine that; every consultation is to be based on, it doesn’t matter if you’re talking to…a mother or a father…it shouldn’t have any significance* [Informant 13]

Regarding whether and how SHD can possibly contribute to a more equal healthcare system, the managers lacked suggestions for this:

*No, this is something each family decides. It’s nothing we can influence; the question is, why would we?* [Informant 7]

The concept Defence was expressed together with other concepts in various ways. For example, in one interview Defence was the only concept in the model that was represented. Further, in three interviews, Defence was displayed together with Denial, and in six cases, together with Denial and Openness. In addition, in four interviews Defence was present together with Openness. Finally, in one interview Defence was displayed together with the remaining three concepts Denial, Openness, and Awareness.

### Openness

The third concept in the conceptual model, Openness, was expressed in 17 of the 23 SHD manager interviews. This concept indicates a person’s readiness to learn more about equity in healthcare and develop competence in this. Hence, it was not all managers who denied the existence of inequity in healthcare, or defended themselves regarding the notion that they were contributing to it. Several managers instead stated that equity is an important issue in healthcare. Examples of expressions connected to this concept included an openness regarding how questions are asked by RNs, and an acknowledgment that gender views in society play a role:

*I think we ask the questions in the same way, but if the answers are different, we come back to the gender perspective in society; if we’re women, we have a harder time standing up for and explaining [what we want].* [Informant 1]

Further examples regarding the managers’ expressions of openness include a wish to raise awareness among their workplace staff regarding how, for example, parents are treated in calls according to gender, and to make a change in (earlier) established treatment patterns:

*Raising awareness…because if you’re not aware of things, I mean, this is what I know and this is what I think I know, and there’s so very much I don’t even know that I don’t know. If you then have a study like this and awareness is raised about it, then you’re also made aware as a telephone nurse and: ‘maybe it is the case that I treat…mothers and fathers differently; maybe it is easier for a father to get an appointment’. These types of things. And if you raise awareness about things, then maybe there’ll also be a change in the pattern you may have created, that’s become some sort of custom or something, either consciously or unconsciously. So that’s how I feel: that raising awareness, that is, conduct these types of studies and research and then raise awareness, and it’s my job as a manager, I believe, to raise awareness.* [Informant 1]

*It’s surely something that has to be done at all workplaces in that case. Do we treat women and men, or mothers and fathers, differently?* [Informant 4]

An explicit awareness of (in)equity was, however, not expressed in this category. Expressing openness rather implied being open to the fact that discrimination can possibly occur and that steps may need to be taken in order to prevent unequal treatment. Further, in interviews in which openness was expressed, the gender norms in society were highlighted, and it was suggested that SHD reflects the prevailing societal discussion climate:

*I think it reflects how we treat each other…our gender approach in society. Unfortunately.* [Informant 6]

The information that male parents making paediatric health calls to SHD had been referred to other health services to a higher extent than female parents had was commented upon, and disturbed managers who expressed Openness:

*It Is that something you carry with you, that you don’t trust the fathers as much as the mothers in taking care of their own child? It almost sounds like it.* [Informant 7]

*It’s troubling; you know, this with heart care, men get much more care than women. It’s troubling to hear this.* [Informant 8]

In one interview, the concept Openness was displayed as the only model concept. In two interviews, it was expressed together with Denial. Further, in six interviews Openness was expressed together with Denial and Defence, while in four interviews it was communicated together with only Defence. There were three interviews in which Openness was solely expressed together with Awareness. In two of these, expressions of Action (further described below) were also included. Lastly, in one interview, Openness was expressed together with the remaining three concepts Denial, Defence, and Awareness.

### Awareness

The fourth concept in the conceptual model, Awareness, was present in four of the 23 SHD manager interviews. This concept points to a person’s consciousness, displayed through expressions of awareness of (in)equity in healthcare. These managers expressed a wish to attain equity in telephone nursing, and that they were keen to work against the discriminatory structures of which they were aware. They commented on inequality between women and men in society, and on how societal norms may impact patient safety in the TN work. The managers promoted RNs’ authority to encourage male parents to call for their children to manage the performance of self-care advice for the child:

*I think it’s a matter of how we treat male/female. Because in cases when you don’t understand, then we’re looking at another aspect, this notion of patient safety…There can’t be interpretation issues, so that you don’t know if you’ve understood…then you have to look at the child. But I think perhaps that it’s not that the fathers don’t understand; more that if there’s the slightest hesitation, that he doesn’t want to…you can sense that in the calls, that he’s hesitant to receive self-care advice; while you perhaps, between two women, might be able to talk in another way, yes, come to some other conclusion. And that you perhaps don’t do this with a man to the same degree.* [Informant 6]

Another manager wondered whether RNs might be influenced by their own life history and act instinctively. It was argued that the typical SHD telephone RN was born around the 40s–50s and raised according to a hetero norm, assuming one possible version of female/male partnership. Further, these managers questioned whether Swedish society has actually become equal, referring to men’s higher wages:

*That type of thing happens unconsciously. It’s the same thing as thinking that… all women have husbands …‘Could I speak with your husband?’ ‘Yes, you can speak with my wife’. It happens unconsciously. But it can very well be the case that you think the men need more help, unconsciously.* [Informant 12]

*We think we’re so forward-thinking, that everything’s shared equally and…everyone does things the same way. But…we were born in the 50s, and some of us in the 40s, it stays with you, unconsciously…the men are the providers [laughs].* [Informant 12]

In addition, one manager reacted with sorrow regarding prevailing healthcare inequalities, such as gendered parental results for paediatric health calls. This manager did not believe there would be a change if the same outcome were measured today or in the near future:

*It’s terrible that it should be that way…I think that today, still, there would be a difference…I think it…would turn out that you are, still, treated differently.* [Informant 14]

Another manager had not previously heard of the unequal female/male parental results for paediatric health calls at SHD. This manager laughed and drew comparisons to other healthcare research, in which men have been found to get access to healthcare before women:

[Laughs] *I haven’t heard about that study, but I’m laughing because I’m thinking: they’ve actually done studies on adults too, where they say it’s easier for men to get care and women have to wait…have longer waiting times and aren’t offered care as easily. So that’s actually the reason I’m laughing at that. I don’t really know…if the study’s like this, then that’s simply the way it is.* [Informant 20]

None of the interviewed managers had Awareness as their only expressed concept. Three of them expressed Awareness together with Openness. In one interview, Awareness was expressed together with the remaining three concepts Denial, Defence, and Openness.

### Action

The fifth concept in the conceptual model, Action, was implicitly present in two of the 23 SHD manager interviews. This concept suggests the ability to perceive challenging situations and judge and act upon them, in order to achieve equity in healthcare. Two managers implicitly expressed a wish for Action. For example, they wanted to raise RNs’ awareness at the workplace and stimulate discussions among their personnel to promote equitable healthcare:

*I think, just by talking, discussing and making people aware…The same as that you can’t say ‘Could I speak with your husband’…You have to really be aware that things today don’t look the same as they did in the past. No. No, but I think so, do things consciously and a reflection, group work of some type, or, yes. Do you have the TRIHS guidelines for telephone nurses? There’s a lot in there. One section on healthcare and supporting equal care and preventing ill health. So you try, but these calls aren’t long.* [Informant 12]

*I’d think it would be a very interesting group project, where…a lot of the telephone nurses would get some food for thought. I don’t think this is really something you think about, it just ends up being that way. So, I think it would be very exciting and would lead to good discussions, raise awareness. Someone’s said that you discuss symptoms among men* vs. *among women.* [Informant 14]

In the two interviews in which Action was implicitly expressed, the concepts Openness and Awareness were also present. In two other interviews with Openness and Awareness present, however, Action was not expressed. In one of these all remaining four concepts were present, while in the other Openness and Awareness were included.

## Discussion

The aim of this study was to investigate SHD managers’ views on gender (in)equity in healthcare through the application of a conceptual model, developed based on empirical SHD telephone RN data [37] as a baseline measure at SHD’s national implementation. To our knowledge, there are no other tools available to asses gender (in)equity in healthcare. The conceptual model was developed by the authors [37] and includes the concepts Denial, Defence, Openness, Awareness, and Action. A striking finding in this study is that all model concepts proved to be present in the manager interviews. The conceptual model is thus confirmed by the present study. Consequently, this implies that health (in)equity data from RNs performing SHD telephone work are confirmed by data from SHD mangers, who supervised and controlled the telephone RN work [38, 39] at SHD’s national completion. Whether manager expressions of Action regarding equitable healthcare at SHD have increased, will be verified in forthcoming measures.

In line with the conceptual model [37], Denial and Defence were often expressed together, and are thus confirmed as a separate block. Likewise, Awareness was often expressed in relation to Openness, mirroring the other block in the model. The two blocks are visualized in Table 2. An important result of the analysis was that Denial and Defence were expressed to a higher extent (14 and 15 interviews, respectively) than Openness and Awareness (17 and 4, interviews respectively). Hence, Denial and Defence were mentioned in 29 interviews, compared to Openness and Awareness, which were mentioned in 21 interviews (see Table 3).

**Table 2:**
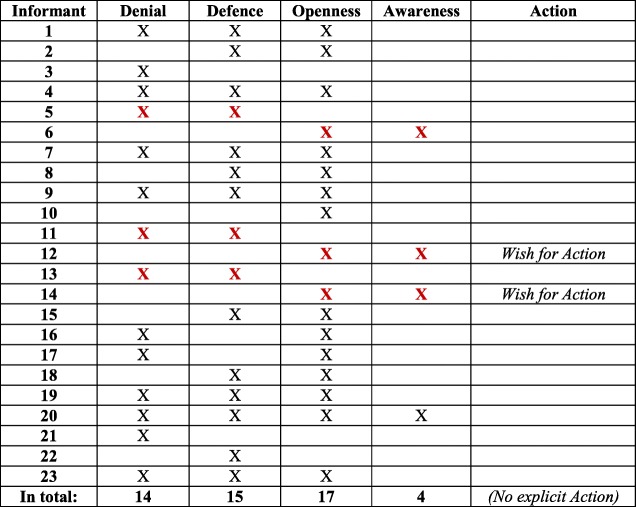
Six informants expressed concepts on either side of ‘The dichotomized two blocks

**Table 3:**
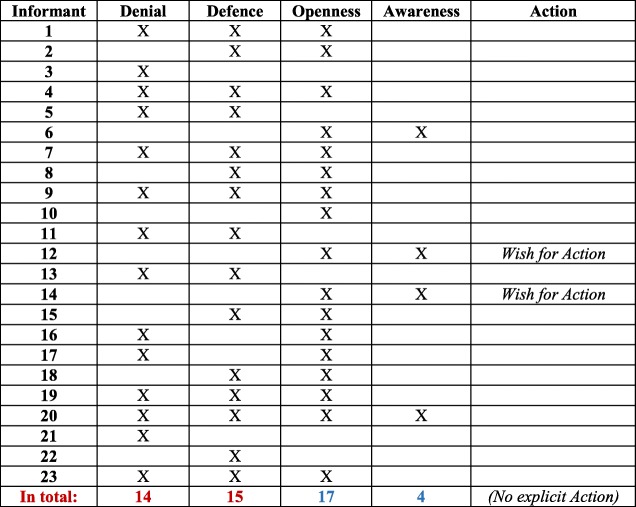
Summarized: 29 versus 21 expressed concepts for ‘The dichotomized two blocks’

Regarding Denial, the SHD managers communicated that healthcare is provided equally and that inequity in health is not a problem, at least not in telephone nursing. This is in line with SHD telephone RN expressions of Denial in Höglund et al. [37]. The same was found concerning the managers’ expressions of Defence, in that working routines, e.g. using the mandatory computerized DST, was assumed to prevent unequal treatment and protect from inequities. The managers’ arguments are not in accordance with the Swedish Healthcare Act, 2017:30 [2]. The law not only prescribes *good health and care on equal terms for the whole population*, but also that *care should be given according to needs*, which seems to sometimes be forgotten in healthcare. Hence, SHD callers with greater needs should *not* be treated the same as other callers but instead be given extra, in a presumably longer call, to achieve the same level of health as for the average citizen.

Concerning Openness, several managers stated (in)equity as an important issue in healthcare, consistent with RNs’ expressions [37]. These managers desired increased awareness of equity among their employed RNs, for example through avoiding old accustomed counselling patterns, such as treating parent callers according to gender. Thereby, SHD will avoid contributing to a traditional form of doing gender [29] and telephone RNs can relate to callers independent of gender. By doing this, they will increase the possibility for all callers to feel included, even those beyond the binary gender norm.

Concerning Awareness, some managers, in line with RNs [37], highlighted the importance of societal work against discriminatory structures for women and men. They were aware of the social construction of women’s gendered identity through women’s responsibility for caring [30, 31, 34], which is also present within Swedish families [23]. Four interviews displayed the two concepts Openness and Awareness together, of which one also contained Denial and Defence; see Table 4. There were, however, few suggestions for Action. The SHD managers rather indicated that a gender-equal caller population is not something SHD can influence [18].

**Table 4:**
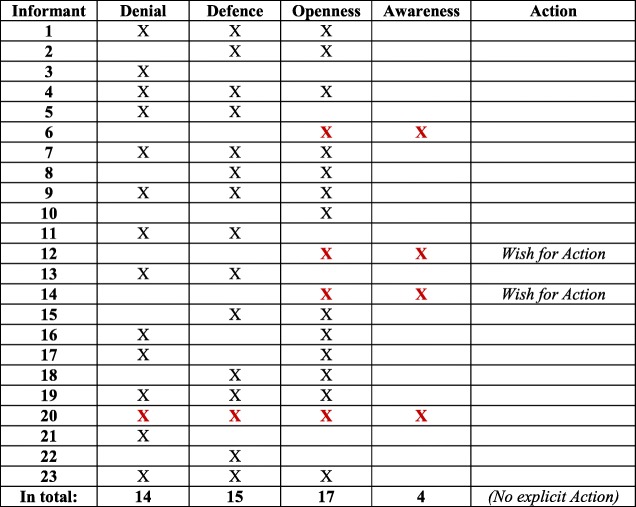
Four informants expressed Openness/Awareness (of whom one expressed *all concepts)*

The concept Action is only implicitly expressed in both RN empirical data [37] and the present manager data. Consequently, neither telephone RNs replying to SHD calls, nor their managers who supervise and control the TN work, explicitly work to promote equitable healthcare at SHD. Two managers suggested discussions and workshops to make SHD telephone RNs more aware of (in)equity. This could be a start, but it is not enough. According to Höglund et al. [35], an educational intervention for SHD telephone RNs, including a web lecture, literature and workshop seminars, revealed some increased awareness of (in)equity in healthcare among the participants. Apart from this, it likely also takes practice to recognize challenging situations and judge and further act upon them, in order to give everyone “a fair opportunity to attain their full health potential” (WHO), i.e. to achieve equity in health.

As mentioned in the introduction, in Höglund et al. [37] the four concepts Denial, Defence, Openness and Awareness were regarded as two qualitatively different blocks, Denial and Defence versus Openness and Awareness, which at the same time are positioned on a continuum. In Table 5, this continuum is shown through the three expressed concepts of Denial, Defence, and Openness. The same line of argument is presented in Table 6, where the two concepts Defence and Openness are expressed. The continuum implies that individuals can move from one position to another, and possibly develop from Denial, via Defence and Openness, to Awareness. Awareness is thus the most desirable of the four, followed by Action as the ultimate position. In four interviews, only *one* of the concepts Denial, Defence, or Openness was present (see Table 7), while Awareness as the only concept expressed in an interview was absent. A noteworthy finding is that Denial was the only concept expressed in two interviews. Finally, two interviews contradicted the idea of blocks/continuum, with the concepts Denial and Openness presented, but lacking Defence in between (see Table 8). This could arguably be seen as an outlier according to the conceptual model.

**Table 5:**
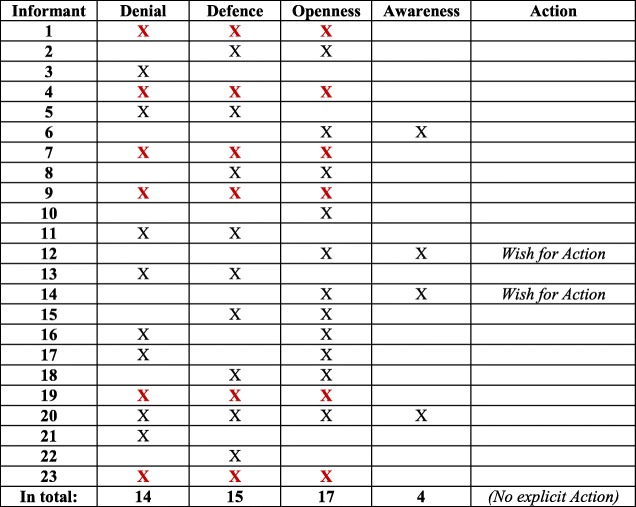
Six informants expressed Denial/Defence/Openness continuum

**Table 6:**
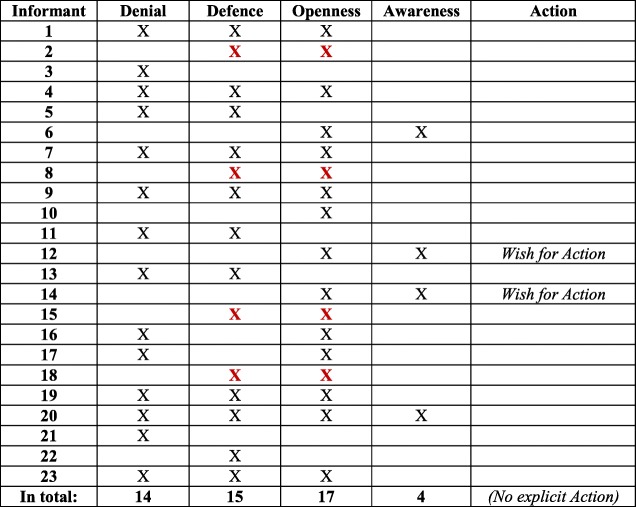
Four informants expressed ‘the middle’ concepts Defence/Openness

**Table 7:**
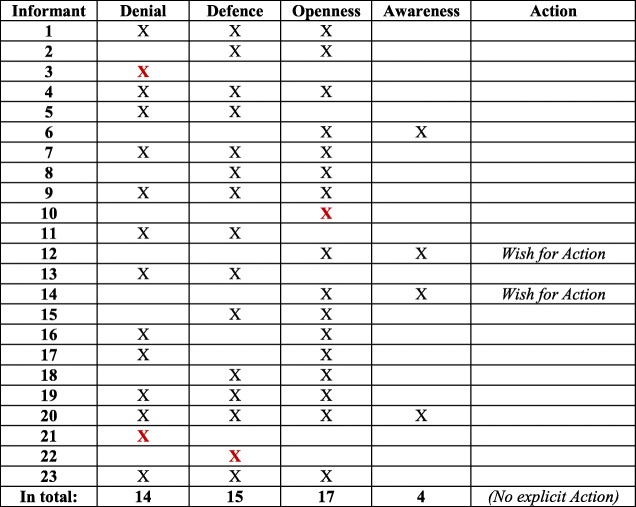
Four informants expressed ‘A single expressed concept’ (none of whom expressed Awareness)

**Table 8:**
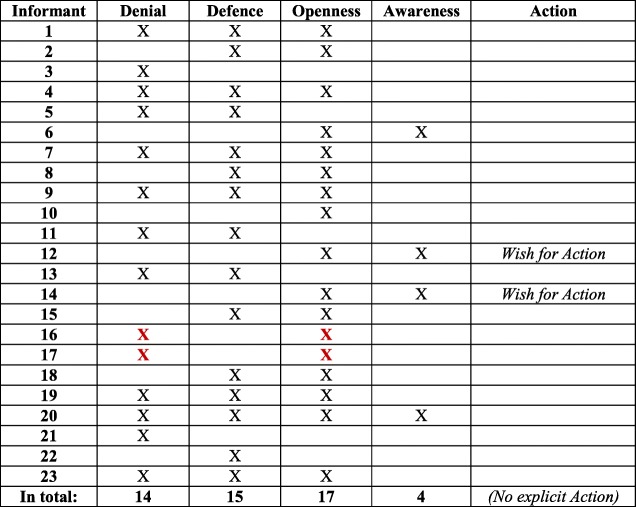
Two informants expressed Denial and Openness – ‘Outliers’?

The results indicate that the interviewed managers, to quite a high extent, lacked awareness of (in)equity in healthcare. However, they did express an openness to learning more. This was also the case among the informants who had expressed Denial and Defence together with Openness. Beyond the presumed model continuum this might reflect a wish to show political correctness, but it also points to the need for educational training in order to increase awareness of equity in healthcare among SHD managers. In a previous study, an educational intervention designed to increase awareness of (in)equity in health has been shown to have impact [35]. This intervention, however, was only directed at SHD telephone RNs. In line with previous studies arguing for increased ethical competence among healthcare staff [44, 45], to be efficient such interventions also need to include the management level.

### Strengths and limitations

The author EK has TN experience while the author ATH does not. This allowed for emic and etic viewpoints and strengthens the study’s confirmability, also established through the presentation of findings at research seminars. Another strength of the study was that *all* employed SHD managers at the time of the study agreed to participate. This increases the transferability of the results by means of substantial data of the whole SHD manager population. It also strengthens the study’s credibility and trustworthiness, together with the transparent description of data collection and analysis process [40]. That fact that only two managers were men, made gender comparisons of the results impossible. The interviews were made in 2012, the year before SHD’s nationally completion and thus constitute a baseline measure. Hence, the data will enable future investigations concerning telephone nursing as a tool for reaching good health and care on equal terms for the whole population [1] as stated in the Swedish Healthcare Act, 2017:30 [2]. Although the data was collected some years ago, the results can be of use for policymakers, e.g. in intervention studies, in order to assure equity policy implementation. The presentation of the research process and findings exemplified by quotes support dependability. It is important to note that the data are self-reported, and do not describe how the managers actually perform their leadership at SHD.

## Conclusion

All model concepts – Denial, Defence, Openness, Awareness and Action [37] – were present in the SHD manager data. However, the last concept – Action – was more implicit, in line with the empirical SHD telephone RN data upon which the conceptual model was built. We thus conclude that neither telephone RNs replying to SHD calls, nor SHD managers who supervise and control the telephone work, explicitly worked to promote equitable healthcare at SHD. In a future study, we plan to use the data of this study as baseline. The two managers’ suggestions regarding Action, with discussions and workshops to make employees more aware of (in)equity, can be a start; but experience and practice are also needed in order to be able to work for equity in healthcare. As managers play a key role regarding SHD telephone RNs’ work situation, educational interventions must also reach the management level if they are to have an effect. Thereby, “action” in the conceptual model can be achieved, and policy implementation to a higher extent may be reached.

## Data Availability

Data have been handled confidentially. Interviews and transcripts are kept in a locked safe at the department.

## Abbreviations

DST: Decision support tool
RN: Registered nurse
SHD: Swedish Healthcare Direct
TN: Telephone Nursing
